# Impaired chair-to-bed transfer ability leads to longer hospital stays among elderly patients

**DOI:** 10.1186/s12877-019-1104-4

**Published:** 2019-03-21

**Authors:** Milene Silva Ferreira, Fabio Gazelato de Melo Franco, Patrícia Silveira Rodrigues, Vanessa Maria da Silva de Poli Correa, Sonia Teresa Gaidzakian Akopian, Gabriel Grizzo Cucato, Raphael Mendes Ritti Dias, Maysa Seabra Cendoroglo, Carolina Nunes França, José Antonio Maluf de Carvalho

**Affiliations:** 1 Hospital Israelita Albert Einstein, Alameda dos Jurupis 777, apartment 242, Sao Paulo, CEP 04088001 Brazil; 2 Universidade Federal de São Paulo, Sao Paulo, Brazil; 3 Universidade Santo Amaro, Pós Graduação em Ciências da Saúde, Sao Paulo, Brazil

**Keywords:** Elderly, Length of stay, Predicting model, Predictors, Rehabilitation

## Abstract

**Background:**

The study objectives were to identify the main predictive factors for long hospital stays and to propose new and improved methods of risk assessment.

**Methods:**

This prospective cohort study was conducted in the clinics and surgical wards of a tertiary hospital and involved 523 elderly patients over 60 years of age. Demographic, clinical, functional, and cognitive characteristics assessed between 48 and 72 h after admission were analyzed to investigate correlations with lengths of stay greater than 10 days. Univariate and multivariate analyses were performed, and in the final model, long-term probability scores were estimated for each variable.

**Results:**

Of the 523 patients studied, 33 (6.3%) remained hospitalized for more than 10 days. Multiple regression analysis revealed that both the presence of diabetes and the inability to perform chair-to-bed transfers (Barthel Index) remained significant risk predictors. Diabetes doubled the risk of prolonged hospital stays, while a chair-to-bed transfer score of 0 or 5 led to an eight-fold increase in risk. **Conclusions:** In this study, we propose an easy method that can be used, after external validation, to screen for long-term risk (using diabetes and bed/chair transfer) as a first step in identifying hospitalized elderly patients who will require comprehensive assessment to guide prevention plans and rehabilitation programs.

**Electronic supplementary material:**

The online version of this article (10.1186/s12877-019-1104-4) contains supplementary material, which is available to authorized users.

## Background

Among hospitalized patients, the geriatric population is the most vulnerable to adverse events. Somella et al. showed that apart from age (> 65 years), the main factors related to adverse events are female sex; admission to hospital emergency, surgery, or intensive care units; and length of hospital stay [1].

The identification of risks must be the first step in the prevention of adverse events related to prolonged hospitalization among elderly patients [2]. In a systematic review, Shepperd et al. found that structured early and postdischarge rehabilitation planning can significantly reduce the length of hospital stays and the associated consequences [3].

Longer hospital stays have been associated with functional loss, increased mortality, readmission rates, and institutionalization among the elderly [4]. The rate of readmission is mainly influenced by risk factors such as the use of seven or more medications, reduction of 56 points or more on the Barthel Index, and hospital stays longer than 13 days. This last variable leads to a two-fold increase in the risk of readmission [2]. Other studies show that approximately 35% of the elderly develop functional loss during hospitalization and that this loss relates directly to the length of stay [5, 6]. Martone et al. (2017) [7] evaluated the development during hospitalization of sarcopenia in older individuals without the disease at hospital admission. The authors found that 15% of these patients developed sarcopenia and that time spent at rest in bed and baseline disabilities are important factors contributing to the onset of sarcopenia. Additional authors have highlighted the importance of a multifaceted evaluation that considers not only clinical but also functional, cognitive, and social factors to identify the risks accompanying prolonged hospitalization. However, there is still a need for validated tools or methodologies that will facilitate the identification of risks in clinical practice [8–10].

With an ever-increasing aging population, the costs related to adverse events due to longer hospital stays rise not only for the individual patient but also for the health system at large. Thus, recognizing the risk factors for longer hospital stays as early as possible is essential for better treatment planning as well as for optimal use of resources [11]. Thus, the aim of this study was to identify the main predictive factors for long hospital stays to improve the process of risk assessment.

## Methods

This was a prospective cohort study involving 523 patients admitted to the clinical and surgical wards of a tertiary hospital. The randomization was made based on the number of discharges, in order to create a proportionality, without selection bias in relation to the diagnosis, since in this hospital the wards are divided by specialty (Neurology, Cardiology, Orthopedics, Surgery, Oncology). Elderly patients (> 60 years of age) admitted to the clinical and surgical wards of this hospital were eligible to be included in the study. Even patients who were unable to respond to the questionnaire were included and the inability to respond to the questionnaire was one of the factors evaluated. Only hemodynamically unstable patients under intensive care and semi-intensive units were excluded.

The variables analyzed were: age, sex, schooling, living alone, institutionalization; previous diagnosis of stroke, or presence of chronic obstructive pulmonary disease, cancer, acquired immune deficiency syndrome, end-stage renal disease, dementia, diabetes, congestive heart failure, liver disease, coronary artery disease and anemia; number of medications taken, number of hospital admissions in the last six months and in previous years; history of falls, recurrent falls; delirium, urinary incontinence, nutritional risk, decrease in level of consciousness, swallowing difficulty, risk or presence of pressure ulcers; Barthel Index score [12] as a measure of functional capacity 30 days before admission and at the time of evaluation; the difference between the two Barthel scores; and cognitive ability evaluated by the Short Portable Mental Status Questionnaire (SPMSQ) [13]. In addition to the total scores of the Barthel scale and SPMSQ, each of the questions contained in these evaluation instruments was also used as a variable.

To avoid overrepresentation of a specific ward with a high rotation of patients (e.g., surgical ward), the sample was divided according to the historical proportion of elder occupancy by ward. As a result, this sample seems to represent the hospital occupancy assuming that each ward contributed a proportionate sample.

Between 48 and 72 h after admission, patients were assessed using the Barthel Index and the SPMSQ. Concomitantly, information concerning the remaining variables was extracted from medical records. Patients were followed until discharge. Hospitalization greater than 10 days was considered a long hospital stay and was analyzed as an outcome. Univariate and multivariate analyses were performed, and in the final model, long-term probability scores were estimated for each of the model variables.

Length of stay and long stay were considered quantitative and qualitative characteristics, respectively, and are presented as the mean and standard deviation; the median, minimum, and maximum; and as absolute and relative frequencies. The individual associations of each variable with length of stay and the estimated odds ratios were calculated using univariate logistic regression with 95% confidence intervals.

A multiple logistic regression model was used to calculate estimates for the variables influencing long-term stay. A stepwise selection method with backward likelihood ratio criterion for selecting the model variables was used with significance levels of 0.05 for input and 0.10 for output.

The receiver operating characteristics (ROC) curve was constructed considering the high-risk population and its outcomes (hospital length > 10 days).

Firstly, the sample size was calculated according to the hospital prevalence of 15% of patients with prolonged length of stay (more than 10 days). Supposing a confidence of 95% and precision of 3%, the number of patients considered to be included in this study was 544. However, after the inclusion of 523 patients, we calculated the sample size again, based on a new prevalence of 6.3% of hospital patients with a prolonged length of stay, assuming a confidence of 95% and precision of 2.08%.

## Results

Table 1 presents the description of the population.

**Table 1:** Description of the population

Variable	
Age, mean (SD)	75.1 (9.6)
Sex (% female)	44.70
Education (%)	
Elementary school	11.90
High School	27.30
University	60.80
Number of diagnosis, mean (SD)	3 (1.4)
Number of drugs, median (IQR)	5 (0;26)
Hospital admissions past 6 months, median (IQR)	0 (0;7)
Diabetes (%)	29.10
Cancer (%)	23.10
Coronary heart disease (%)	10.90
Heart failure (%)	9.20
Stroke (%)	7.1
Dementia (%)	6.90
Liver disease (%)	3.80
Chronic Obstrutive pulmonary disease (%)	3.30
Renal failure (%)	2.90
Living alone	17.80
Functional status (Barthel index %)	
Independent	47
Mild dependency	16
Moderate dependency	20
Severe dependency	9
Total dependency	8
Cognitive status (SPMQS %)	
Normal	76.0
Mild cognitive deficit	5.6
Moderate cognitive deficit	2.7
Severe cognitive deficit	0.6
Not able to answer	15.1

Descriptive statistics was performed using frequency distribution. Continuous data were reported as medians and interquarile range (IQR) for non-parametric data or means and standart deviation (SD) for parametric data

Univariate analysis revealed that longer hospital stays were significantly associated with several demographic, clinical, cognitive, and functional variables (Tables 2 and 3). Significant variables included age, stroke, congestive heart failure, diabetes mellitus, dementia, delirium, incontinence, difficulty in swallowing, nutritional risk, decreased level of consciousness, pressure ulcers, anemia, number of medications taken (more than 5 medications), and cognitive and functional profile (all *p* values < 0.05; Table 3).

**Table 2:** Univariate logistic regression, qualitative variables

Variable	Hospital length				Total	OR	CI (95%)		p
	Short< 10 days		Long≥10 days						
	n	%	n	%			Lower	Upper	
Stroke (yes)	27	73.0	10	27.0	37	2.63	1.21	5.70	0.014
Congestive heart failure (yes)	37	77.1	11	22.9	48	2.10	1.01	4.33	0.046
Diabetes Mellitus (yes)	121	79.6	31	20.4	152	2.18	1.30	3.65	0.003
Dementia (yes)	24	66.7	12	33.3	36	3.70	1.76	7.79	0.001
Delirium (yes)	38	69.1	17	30.9	55	3.50	1.85	6.64	< 0.001
Urinary incontinence (yes)	156	80.8	37	19.2	193	2.14	1.29	3.55	0.003
Nutritional risk (yes)	293	84.0	56	16.0	349	2.18	1.18	4.05	0.013
Swallowing deficit (yes)	10	66.7	5	33.3	15	3.41	1.13	10.29	0.030
Decreased level of consciousness (yes)	5	38.5	8	61.5	13	11.56	3.67	36.46	< 0.001
Pressure ulcers (yes)	32	66.7	16	33.3	48	3.90	2.01	7.57	< 0.001
SPMQS^b^									< 0.0001^a^
0-2 correct answers^b^	49	65.3	26	34.7	75				
3–5 correct answers^c^	9	75	3	25	12				
6–7 correct answers^d^	24	80	6	20	30				
8–10 correct answers^e^	370	91.4	35	8.6	405				
Barthel index: 453 87.0			70	13.0	523				
- Grooming						0.77	0.69	0.85	< 0.001
- Bowels						0.86	0.81	0.92	< 0.001
- Bladder						0.89	0.84	0.95	< 0.001
- Feeding						0.83	0.77	0.89	< 0.001
- Toilet use						0.81	0.76	0.86	< 0.001
- Transfer						0.84	0.80	0.88	< 0.001
- Mobility						0.88	0.84	0.92	< 0.001
- Dressing						0.81	0.76	0.87	< 0.001
- Stairs						0.82	0.77	0.88	< 0.001
- Bathing						0.81	0.73	0.90	< 0.001

Results from univariate logistic regression

^a^Pearson Chi-Square

^b^Orientation severely impaired

^c^Orientation moderately impaired

^d^Orientation mildly impaired

^e^Orientation intact

**Table 3:** Univariate logistic regression, quantitative variables

Variable	Mean	SD	Median	Minimum	Maximum
Age (years)	75.14	9.54	74.3	60	109
Number of diagnosis	3.02	1.41	3	1	13
Number of drugs	6.02	3.84	5	0	26
Number of admissions last 6 months	0.73	1.15	0	0	7
Hemoglobin	12.55	1.85	12.7	6.6	17.3

Special attention was given to levels of cognitive impairment; longer hospitalization was associated with lower overall scores as well as low scores on each of the questions on the SPMSQ (*p* < 0.001; Table 2). In addition, lower levels of independence in performing daily life activities (assessed by the Barthel Index) were also associated with prolonged hospitalization (*p* < 0.01). This was true for Barthel scores assessed at two different times both at admission and after 30 days, although they did not differ from each other.

Multiple regression analysis revealed that both the presence of diabetes and the inability to perform chair/bed transfers remained significant predictors of risk (Table 4). The presence of diabetes doubled the risk of prolonged hospital stays, while most importantly, a chair/bed transfer score of 0 or 5 (unable and needs strong help for transferring, respectively) led to an eight-fold increase in risk. Furthermore, while patients with diabetes who were independent had a 10% risk of a prolonged hospital stay, diabetic patients who were not independent had a risk of 48.2% (Table 5).

**Table 4:** Multiple logistic regression

Variable	OR	IC (95%)		p
		Lower	Upper	
Diabetes	1.94	1.11	3.39	0.020
Chair/bed transfer (0/5)	7.96	4.61	13.76	< 0,001

*OR* Odds ratio, *IC* Confidence interval

**Table 5:** Values of probability according to the final model

Diabetes	Chair/bed transfer	Probability of LOS
No	10/15	5.7
	0/5	32.4
Yes	10/15	10.5
	0/5	48.2

*LOS* Length of hospital stay

0: unable; 5:strong help for transference; 10: mild help for transference; independent

Of note, the chair/bed transfer item was more strongly associated with the outcome than was the total Barthel score. The AUC (area under the curve) was 0.773; therefore, this model seems to have good accuracy in identifying high-risk patients. Additional informations are available in the Additional file 1.

## Discussion

In developing countries, such as Brazil, the impact of aging on health services is considerable. As the Brazilian population is aging at a faster pace compared with other countries [14], it is important to identify high-risk patients who would benefit from an intensive approach to address their individual needs.

In this study, longer hospital stays among elderly patients were significantly associated with several variables, including age, stroke, congestive heart failure, diabetes mellitus, dementia, delirium, incontinence, difficulty swallowing, nutritional risk, decreased level of consciousness, pressure ulcers, anemia, number of medications (more than 5 medications increased the risk of longer hospitalization), and cognitive and functional abilities (all *p* values < 0.05; Table 2). In our study, we observed that only diabetes and an inability to perform bed/chair transfers (assessed with the Barthel Index) were independently associated with higher risk. Therefore, we suggest that these two variables may provide the best initial screening to identify patients to receive a later, more comprehensive assessment, which will help them get the proper care needed.

Other studies also reported that diabetes is associated with an increased risk of hospitalization and longer hospital stays [15–17]. In our study, 29% of patients had a diagnosis of diabetes, and of these, 20% had hospital stays longer than 10 days furthermore, diabetic patients had approximately twice the risk of long-term stays compared to nondiabetic patients (odds ratio - OR 1.30–3.65).

Some authors have also implicated functional capacity as a strong predictor of long hospital stays as well as institutionalization and death [18, 19]. For example, frailty markers are associated with adverse health outcomes, both within the hospital and in the community at large. Gait speed could be used as an initial screening for risk of long hospital stays and for home discharge. However, its applicability is limited to patients with testable clinical, physical, and cognitive conditions. Therefore, we believe that assessing bed/chair transfer is more useful as a risk detector in the hospital setting, as it would not exclude a significant proportion of the population [20].

The Barthel Index is an internationally used instrument for functional assessment, and some authors have also found it useful to detect adverse events during hospitalization [10, 12, 21]. In our study, patients who obtained a score of 0 or 5 on the chair/bed transfer item of the Barthel Index had eight times the risk of remaining in the hospital for a longer period (OR 4.61–13.76).

It should be noted that in some studies cited, the average length of stay in the hospital for elderly patients was 10 days (7–14 days) [22], and 28 days was considered the cutoff point for long hospital stays. In contrast, in our study, the average hospital stay was six days, and only 6.3% of our population stayed in the hospital for longer than 10 days (as opposed to approximately 54% in other studies).

The limitations of our study were the relatively small number of elderly patients who were hospitalized for more than 10 days (*n* = 70). This reflects our hospital’s constant concern with the clinical consequences of long hospitalization times and the efforts to reduce them. Additionally, in contrast to other studies, we excluded hemodynamically unstable patients under intensive care and semi-intensive units, because they are prone to longer stays by definition. Furthermore, we hoped to identify other risk factors in our sample, and we considered critically ill patients to be a confounding factor. However, an important feature of our work is that we included elderly patients from all clinical and surgical units of our large general hospital, regardless of diagnosis at admission. The nutritional evaluation was not performed using a standardized tool for elders. However, an experienced clinical nutritionist assessed all elders during the hospital admissions; this would have a good sensitivity to identify elders at risk of undernutrition. Additionally, the number of elders enrolled in this study was slightly lower than estimated number determined by the sample size calculation. However, on ROC analysis, we observed good accuracy (AUC = 0.773) demonstrating good quality of the classification model.

These findings reinforce the need for multidimensional evaluations for hospitalized elderly patients to prevent serious adverse events, as suggested by Ellis et al. [23]. However, recognizing a subset of questions that could potentially identify patients at risk for longer hospitalizations seems important, as those elders would have multidimensional needs for intensive rehabilitation and clinical surveillance. The utilization of geriatric wards would help meet those needs. However, we must identify the patients who would most benefit from this still limited and costly resource.

The goal of this study was to construct an instrument that would predict a specific outcome important for daily practice. In fact, we believe that there might be an overlap of high-risk patients identified by this model and elders with frailty or disability. However, the selection of patients according to simple characteristics, such as diabetes and the inability to transfer, followed by referral to an appropriate model of care, would be necessary to reduce hospital length of stay. Specific rehabilitation and geriatric assessment wards for the population at risk would be necessary to be able to rehabilitate patients at functional/clinical risk. Certainly, a comprehensive geriatric assessment would provide a better estimate of patients’ needs, but it would require time and the training of a team.

## Conclusions

In this study, we proposed an easy method that can be used, after external validation, to screen for long-term risk (using diabetes and bed/chair transfer) as a first step in identifying hospitalized elderly patients who will require a later comprehensive assessment to guide their prevention and rehabilitation programs. This method of evaluation can help organize hospital processes to better define quality of care targets and optimize the use of resources.

## Additional file

Additional file 1: data bank. The file corresponds to the data bank, including every information’s related to this article. (XLSX 247 kb)

## Abbreviations

AUC: Area under the curve
OR: Odds ration
ROC: Receiver operating characteristics
SPMSQ: Short Portable Mental Status Questionnaire
